# Supplementary material for: Systematic cross-sectional age-associations in global fMRI signal topography
Source: Imaging Neurosci (Camb). 2024 Mar 8;2:imag-2-00101. doi: 10.1162/imag_a_00101 (PMC12224465; doi:10.1162/imag_a_00101)

Supplemental Figure 1: Age and sex distribution, the relationship between head motion and age, the relationship between number of volumes scrubbed and age, the relationship between MRI scan start time and age, and the relationship between sleep quality and age.

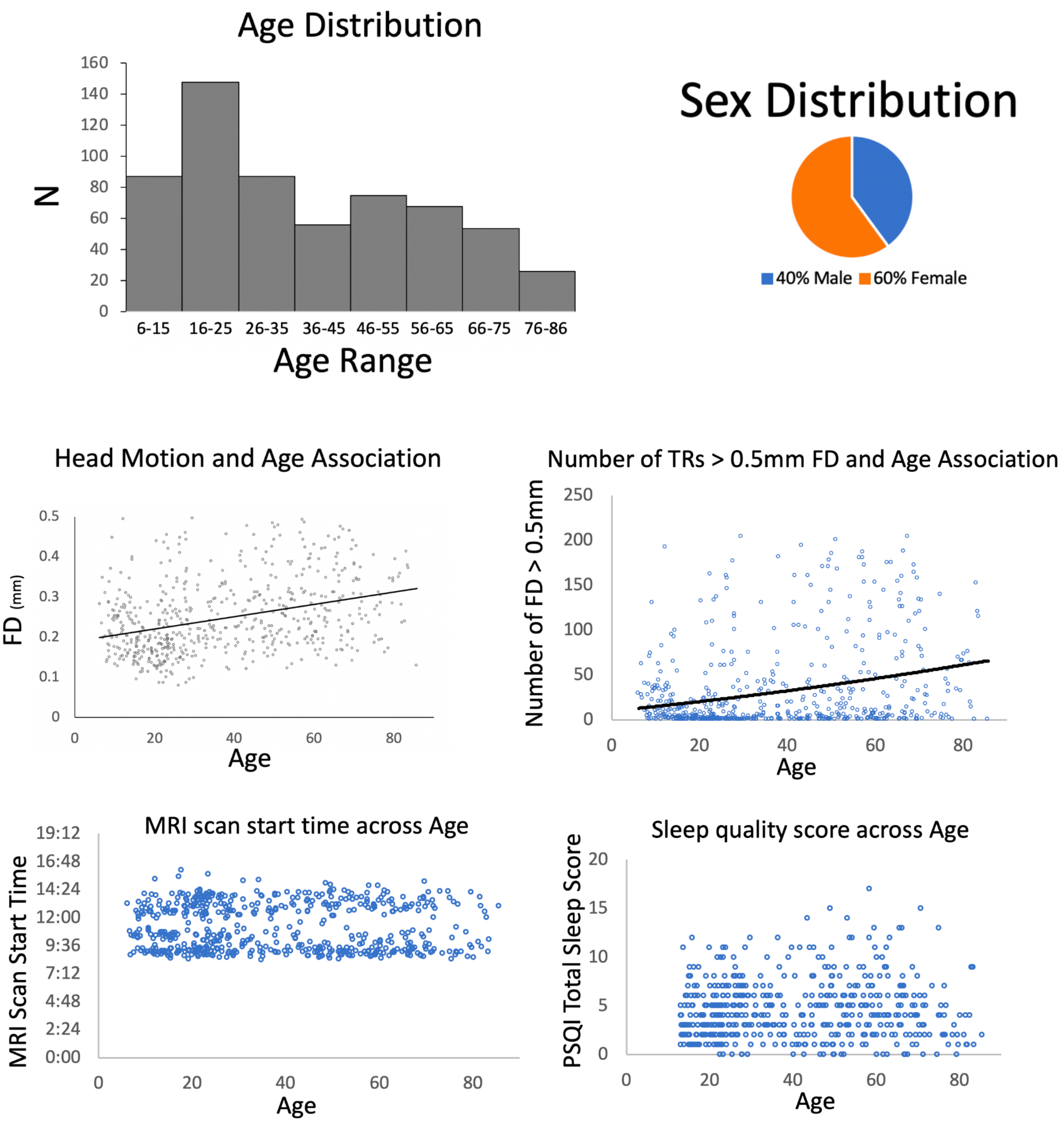

Supplemental Figure 2: Group spatial ICA components classified as signal.

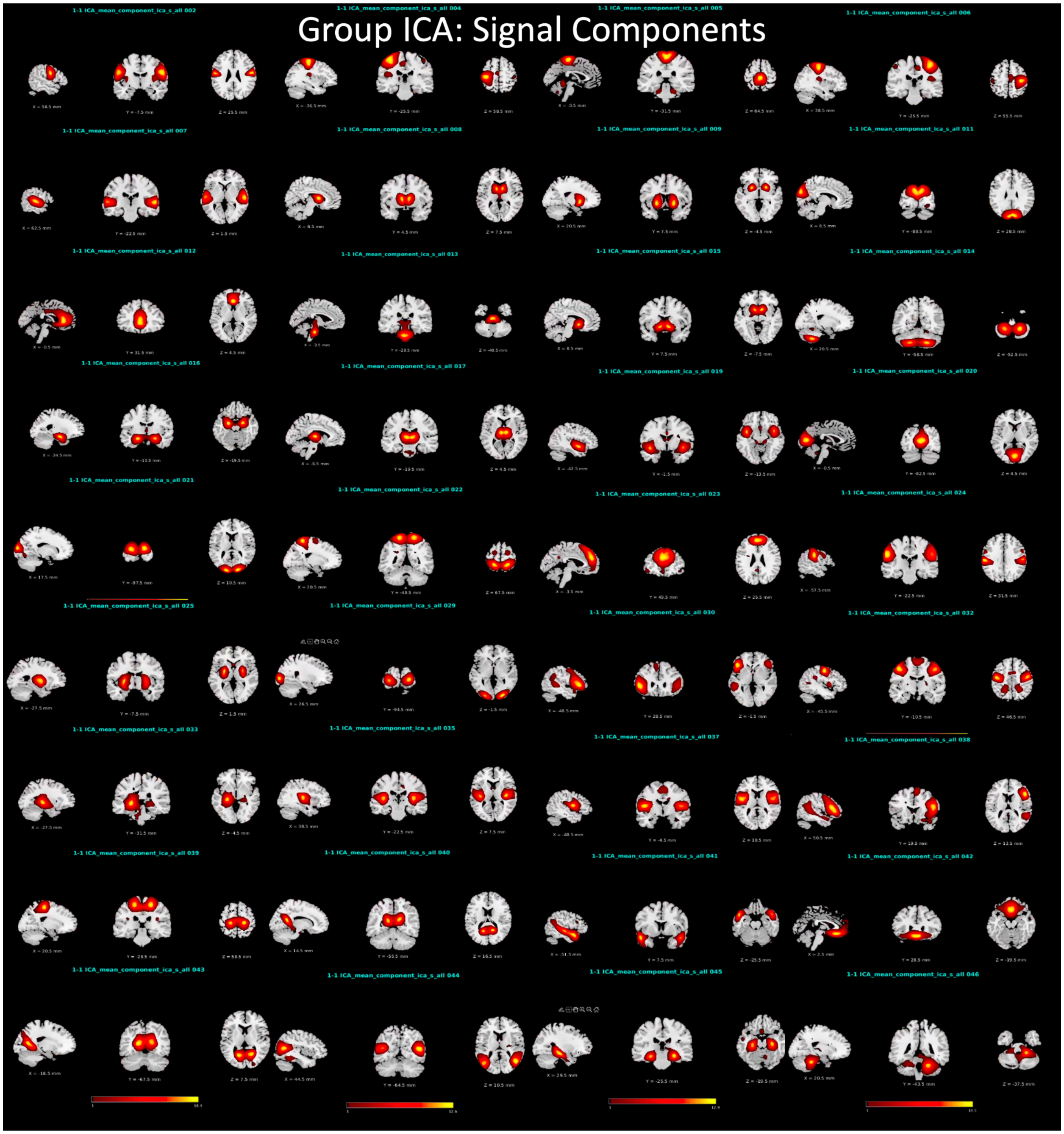

Supplemental Figure 3: Group spatial ICA components classified as signal.

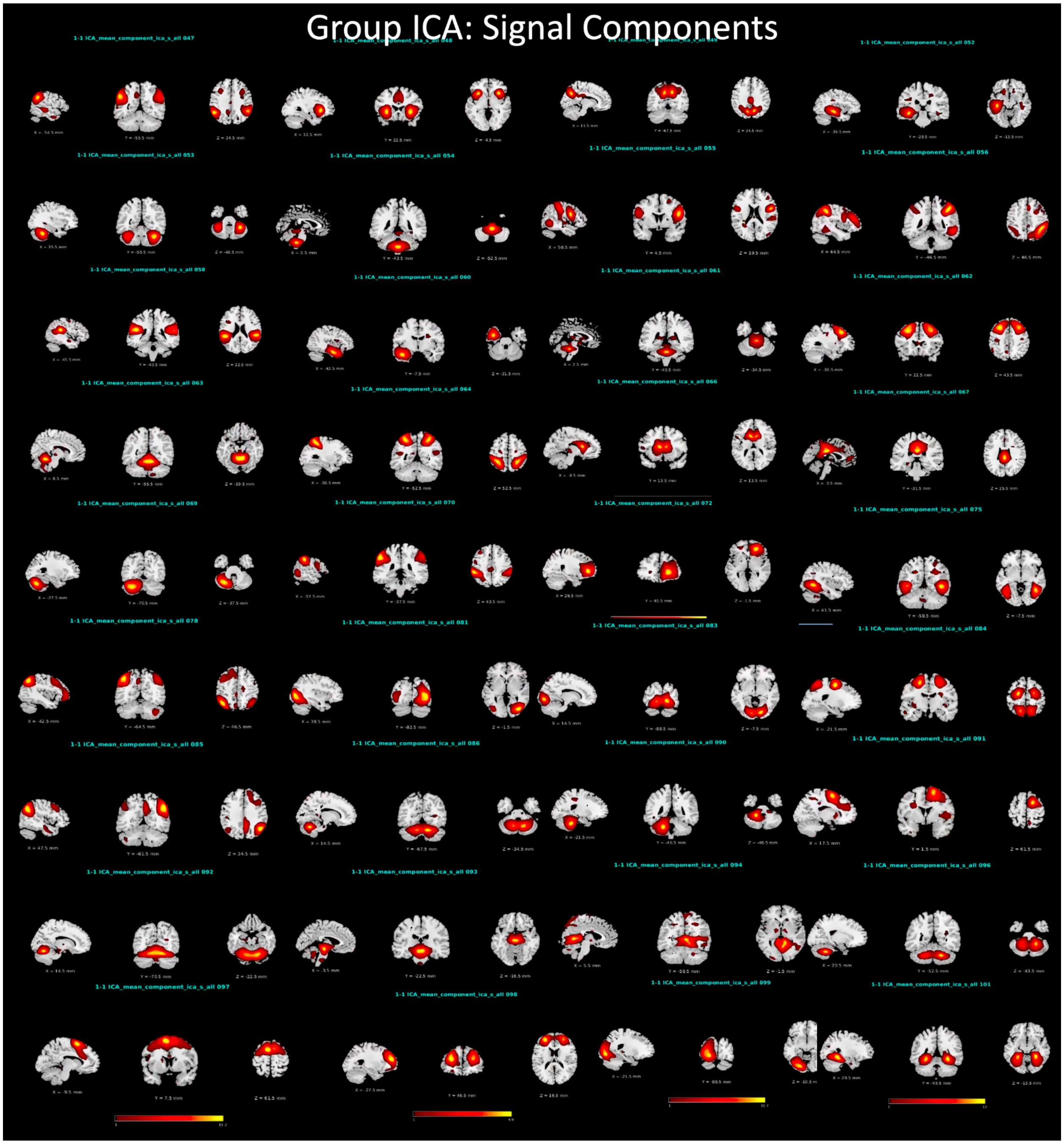

Supplemental Figure 4: Group spatial ICA components classified as signal.

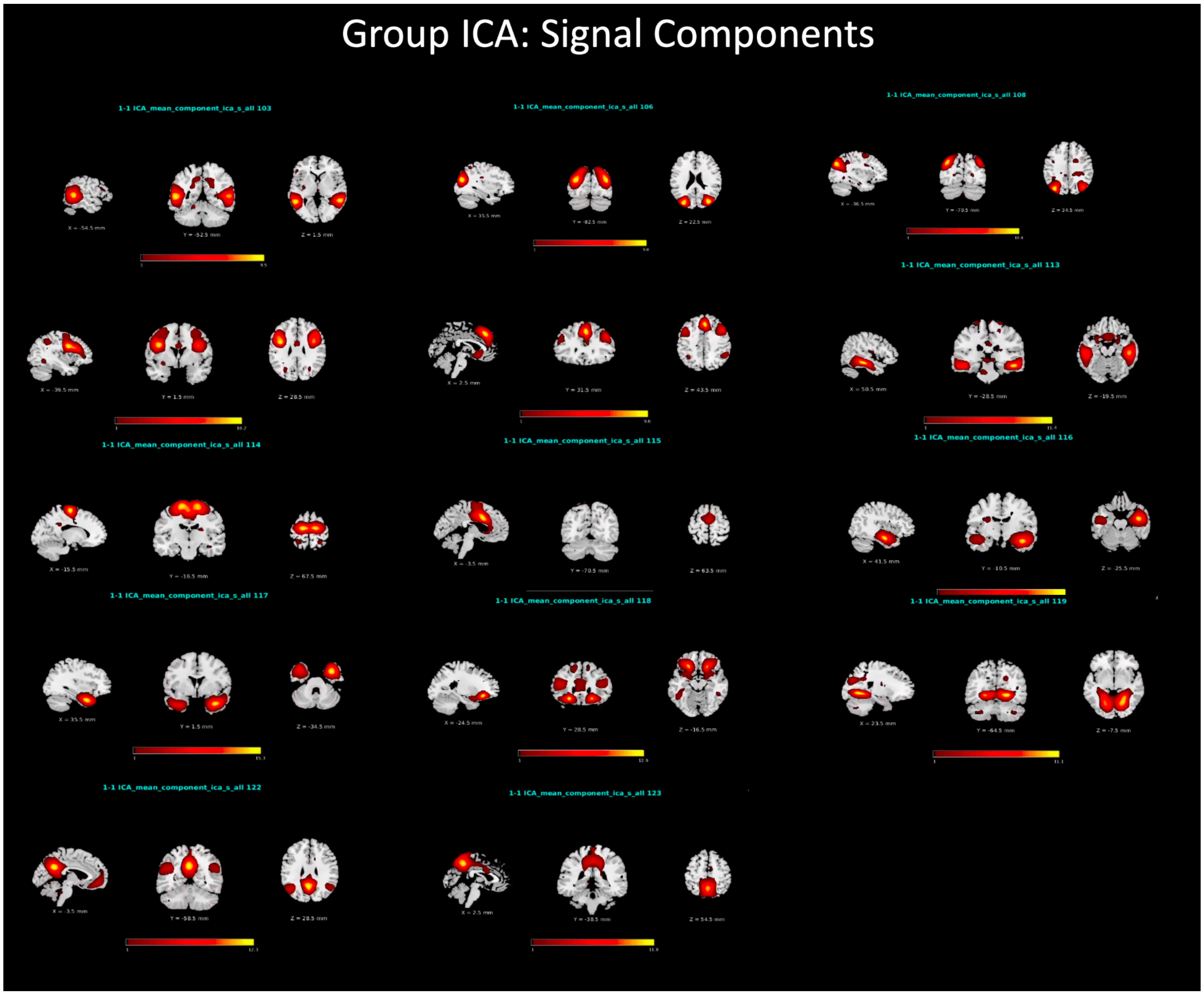

Supplemental Figure 5: Group spatial ICA components classified as noise.

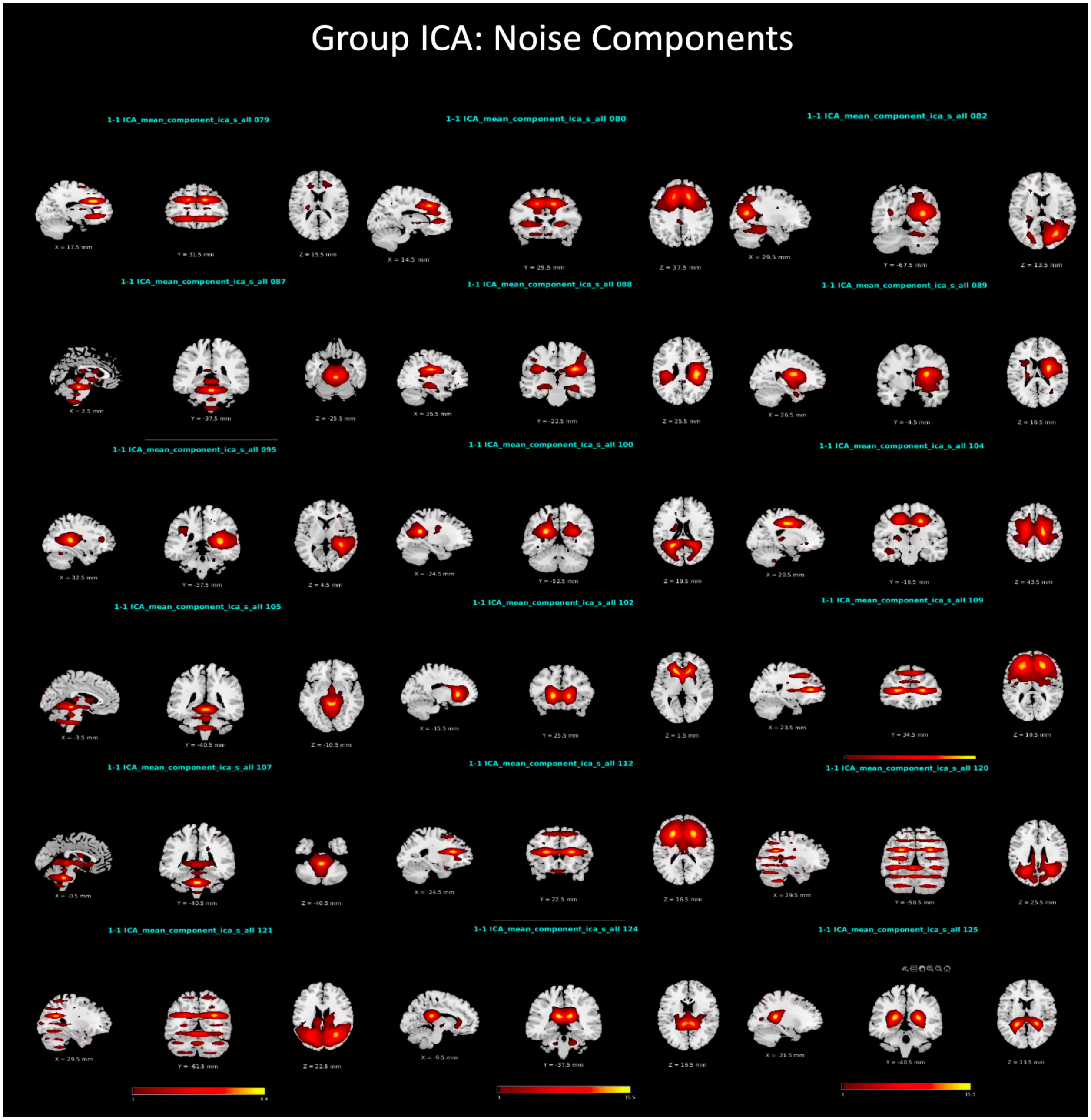

Supplemental Figure 6: Group spatial ICA components classified as noise.

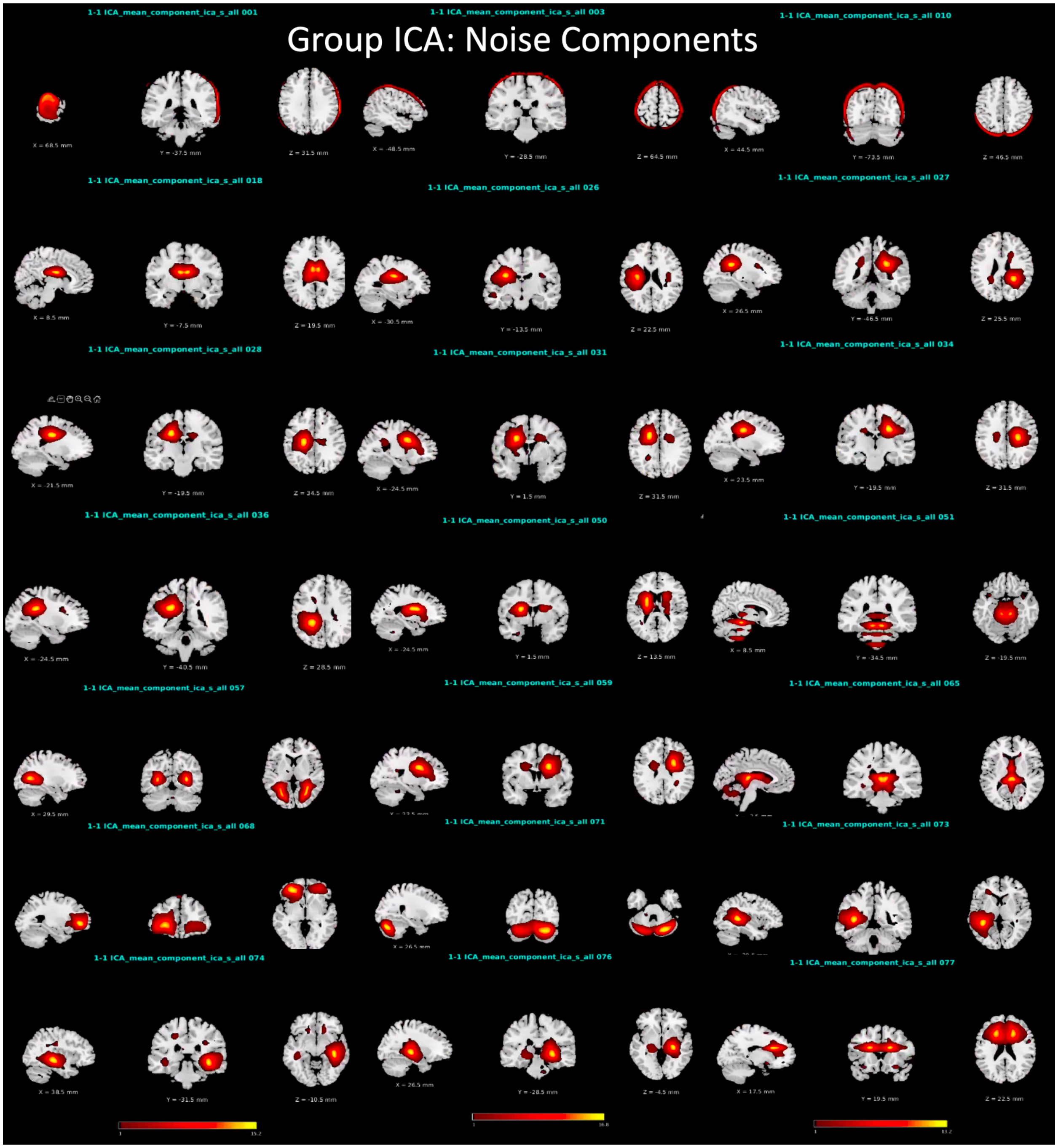

Supplemental Figure 7: tICA components classified as signal shown with positive (hot) and negative (cold) colors.

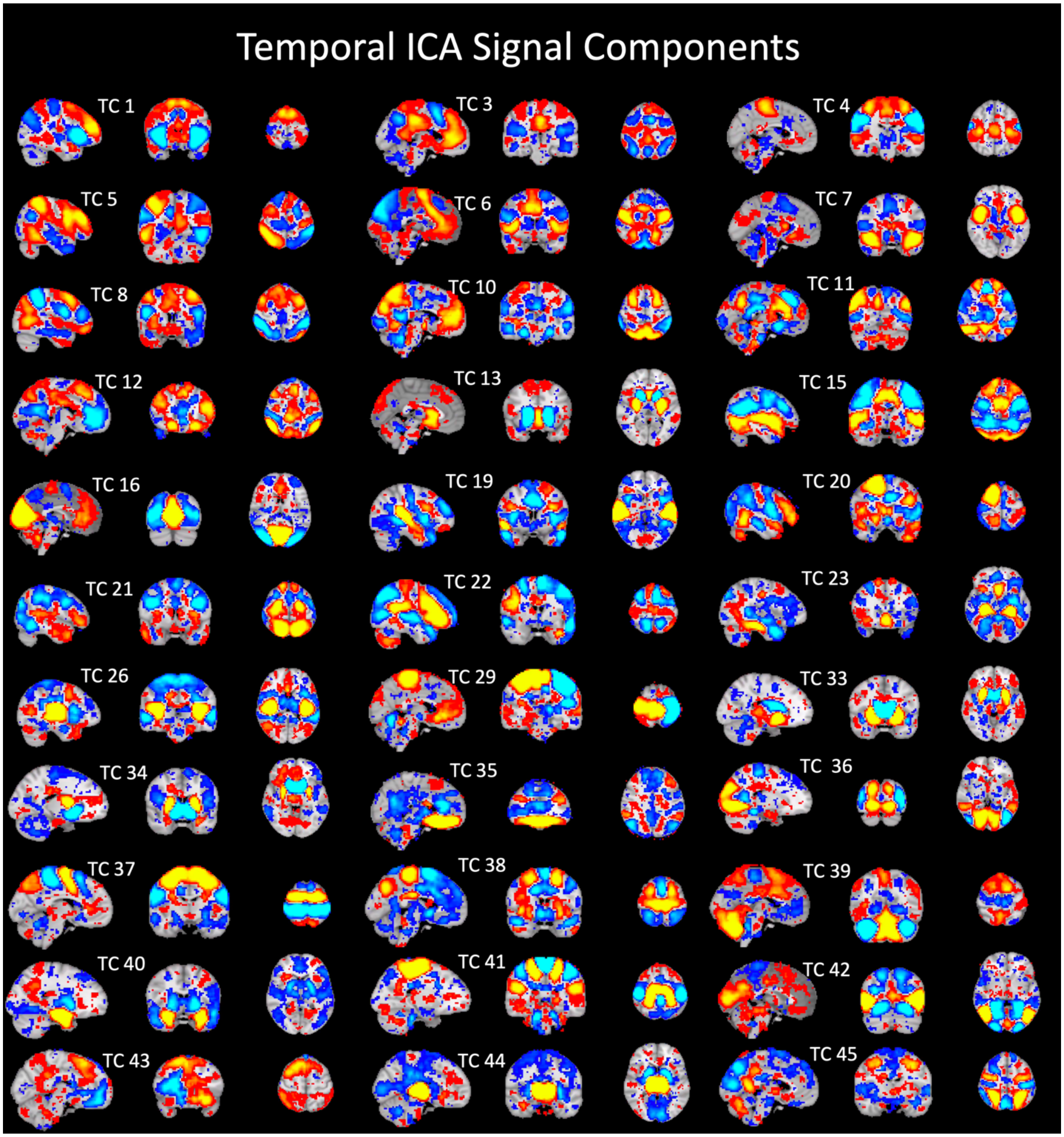

Supplemental Figure 8: tICA components classified as neural signal shown with positive (hot) and negative (cold) colors.

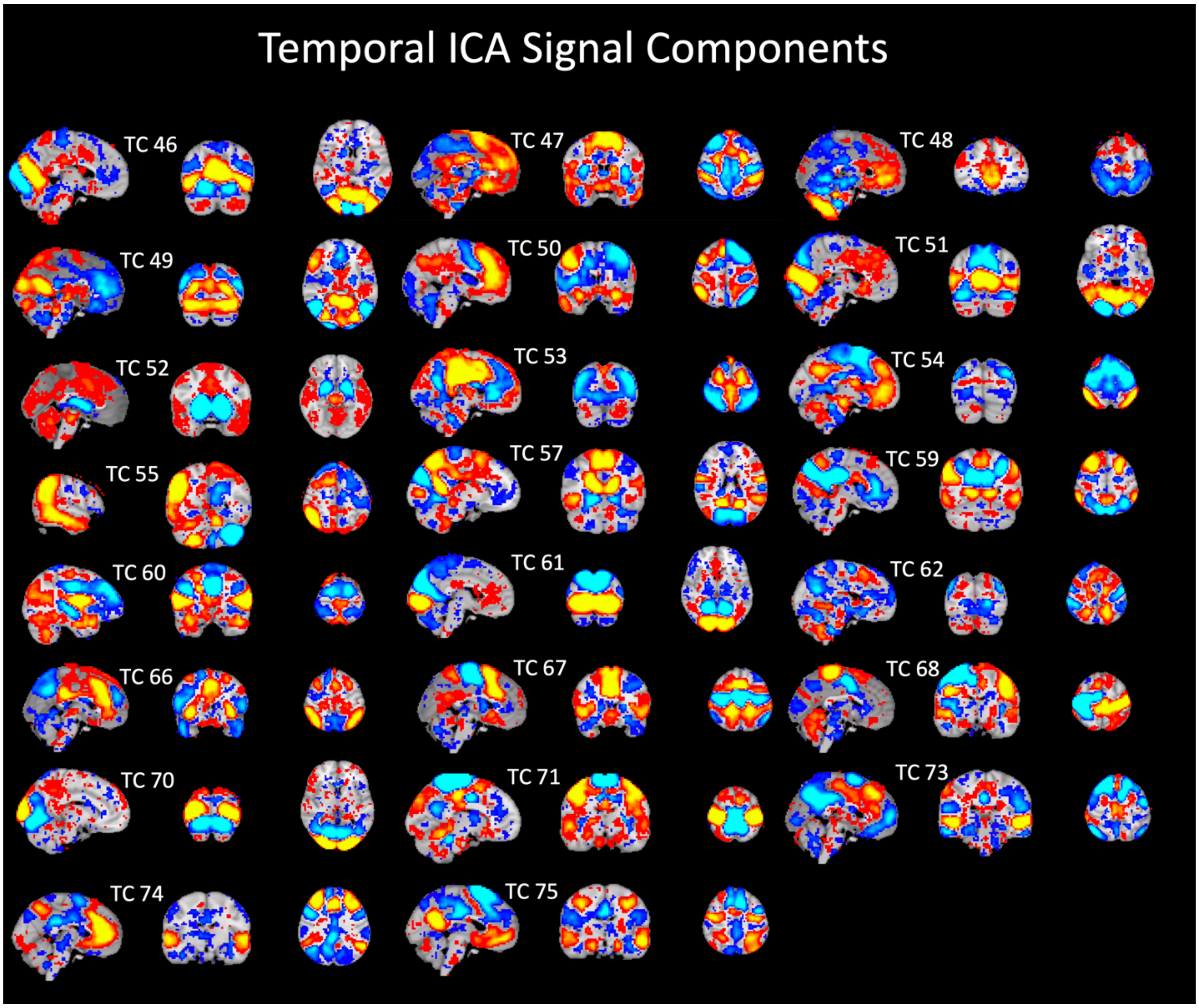

Supplemental Figure 9: tICA components classified as noise shown with positive (hot) and negative (cold) colors. The boxes highlighted in yellow were components used in a more conservative tICA denoising with only 8 tICA noise components. The results were similar to all other pipelines. TC 72 presented a general global reduction across the brain similar to previous studies identifying this component as "global noise" (Glasser et al., 2018; Smith et al., 2012). Pipelines including or not including this component produced similar results.

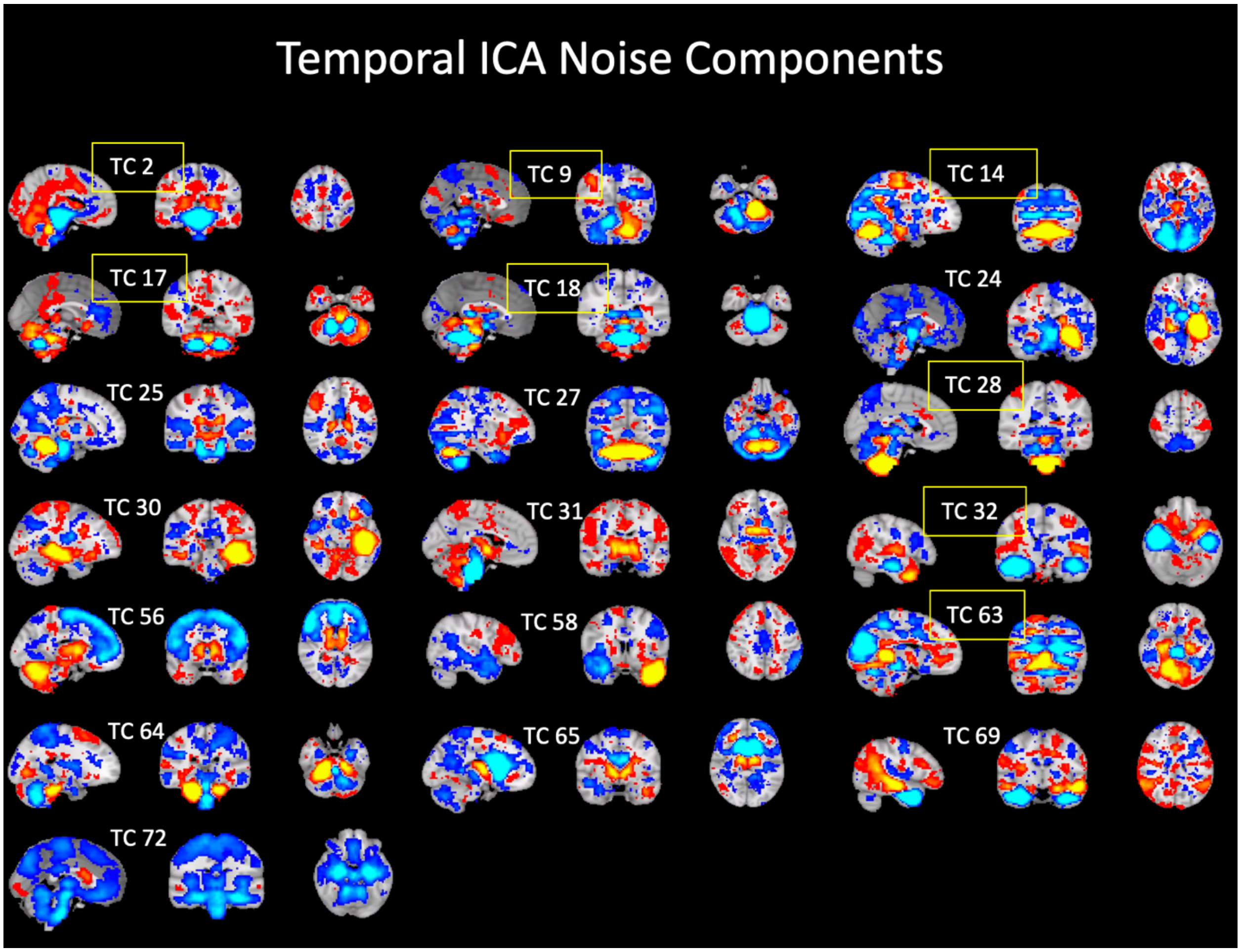

Supplemental Figure 10: Linear and quadratic global signal topography age effect group spatial maps (voxel-wise uncorrected ($p < 0.001$) and cluster-wise corrected ($p < 0.05$). These spatial maps show the consistency of the global signal age topography effects across preprocessing pipelines. The tICA figures are the same images from main Figure 3 and are presented here for comparison.

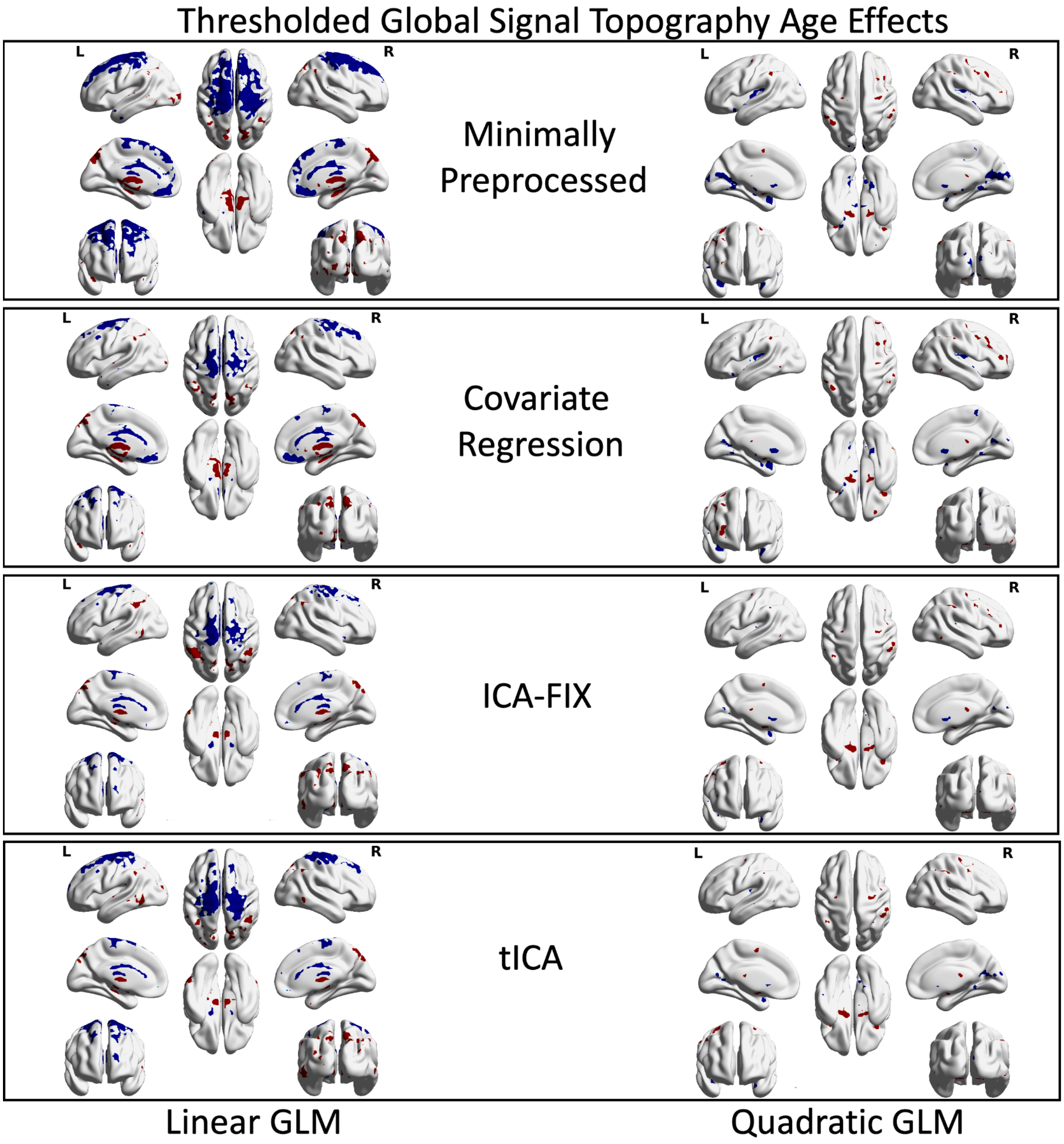

Supplemental Figure 11: Unthresholded linear and quadratic global signal topography age effect group spatial maps. These show the consistency of the global signal age topography effects across preprocessing pipelines.

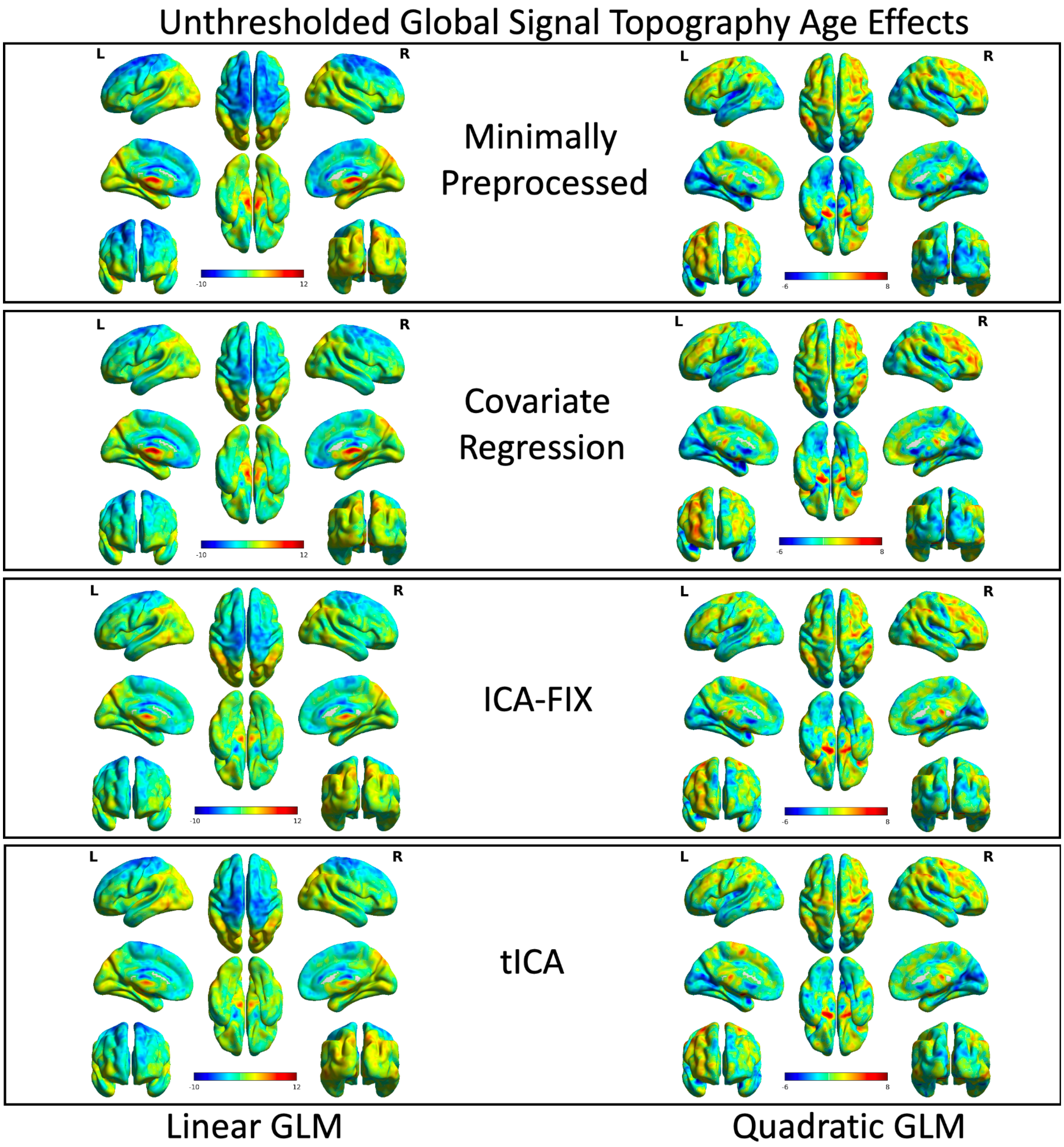

Supplemental Figure 12: Linear and quadratic global signal topography age effect group spatial maps with no volume scrubbing applied (voxel-wise uncorrected ($p < 0.001$) and cluster-wise corrected ($p < 0.05$). These spatial maps show the consistency of the global signal age topography effects across scrubbing pipelines from Supplemental Figure 10 and non-scrubbing pipelines.

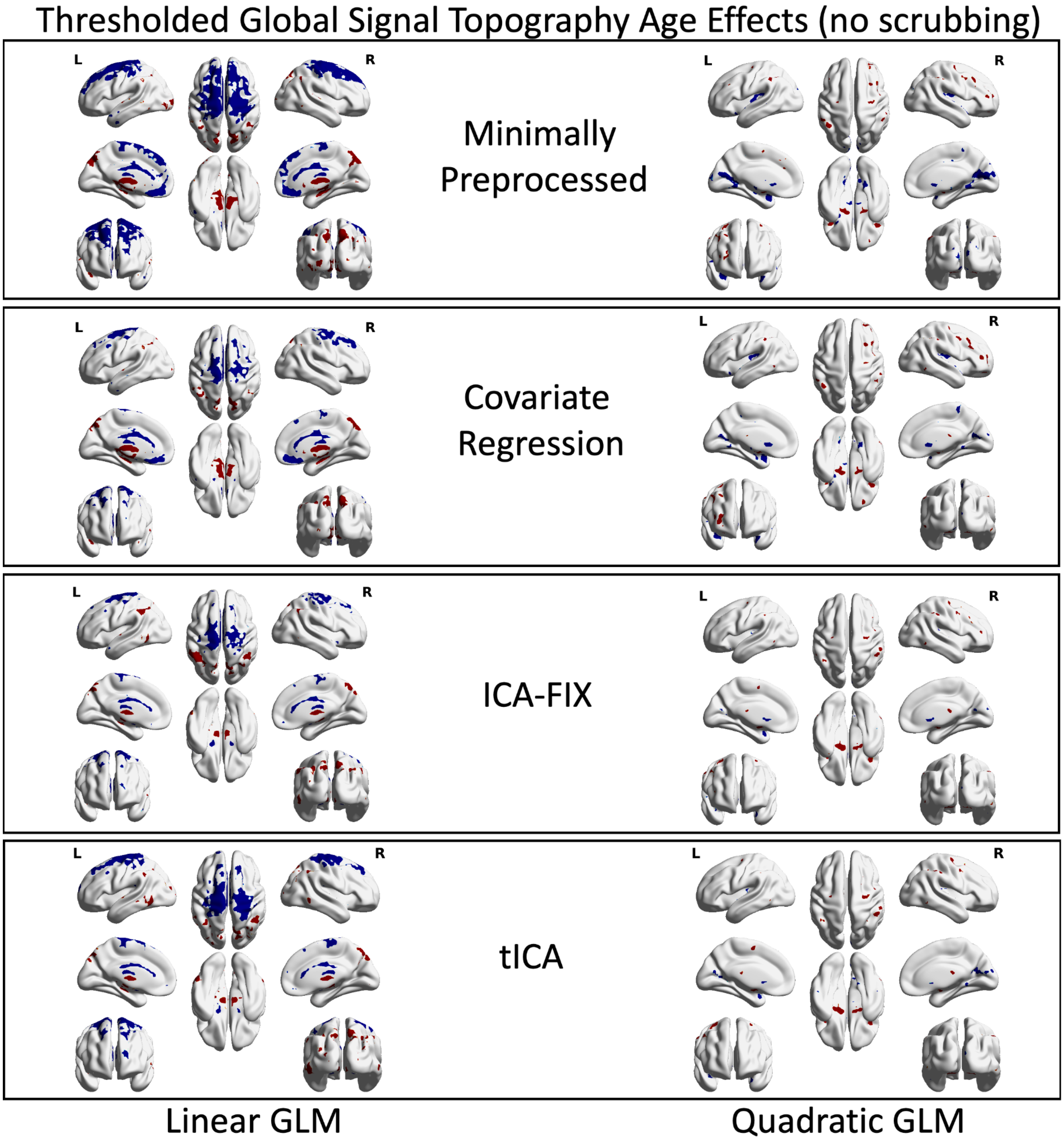

Supplemental Figure 13: FD-FC scatter plots showing the relationship between head motion and functional connectivity strength relative to the euclidean distance between ROIs from the Schaefer 400 17 network parcellation. The scatter plots show no distance dependence effect of head motion on FC strength.

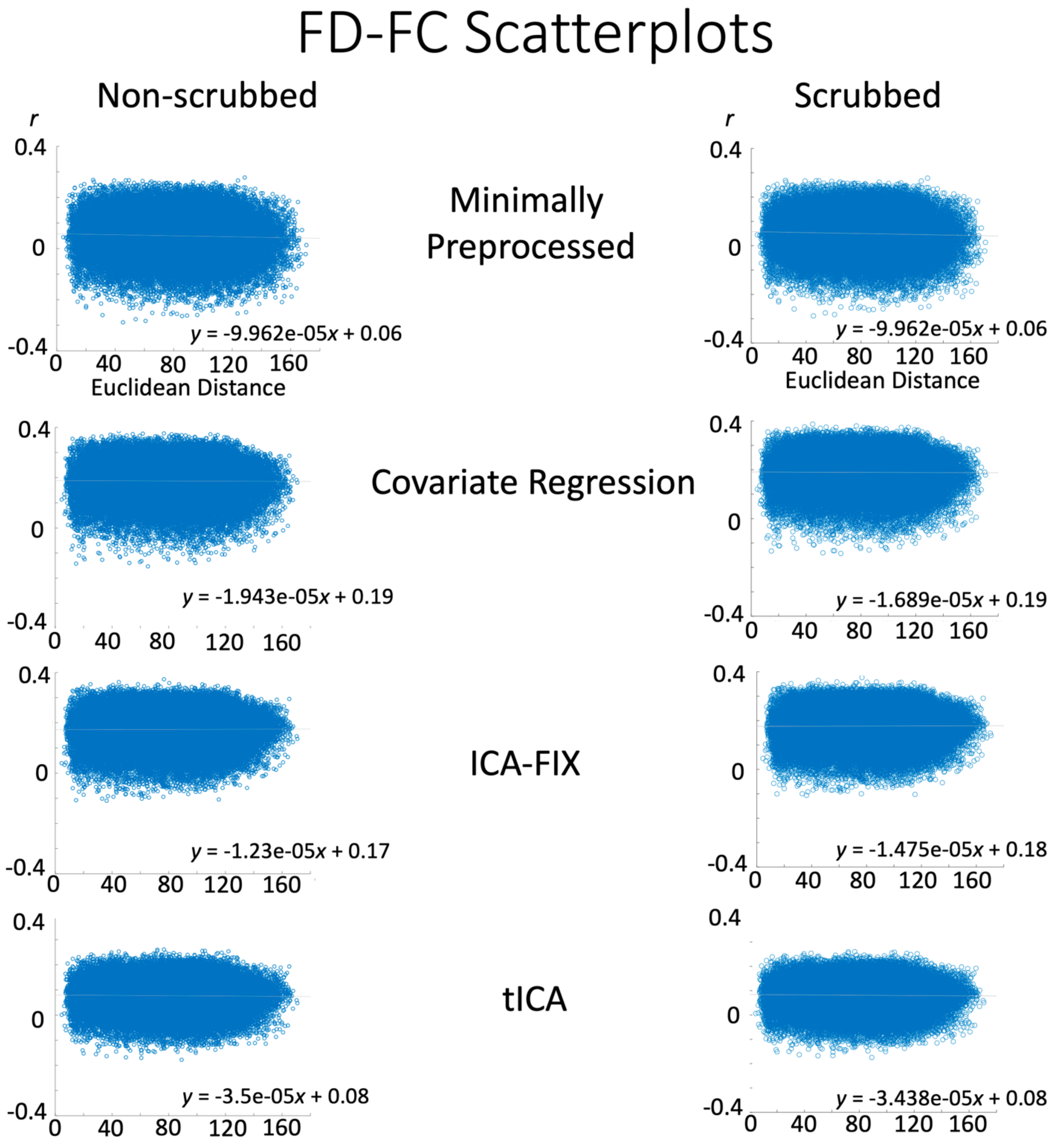

Supplemental Figure 14: The multivariate voxel-wise spatial relationship between GS topography and head motion. A PLS analysis was conducted on individual subject GS topography spatial maps from the tICA preprocessing pipeline using framewise displacement as a behavioral head motion covariate of interest. Colors represent bootstrap ratios that approximate z values (thresholded at approximately $p < 0.001$); warmer colors represent positive associations with head motion while cooler colors represent negative associations with head motion.

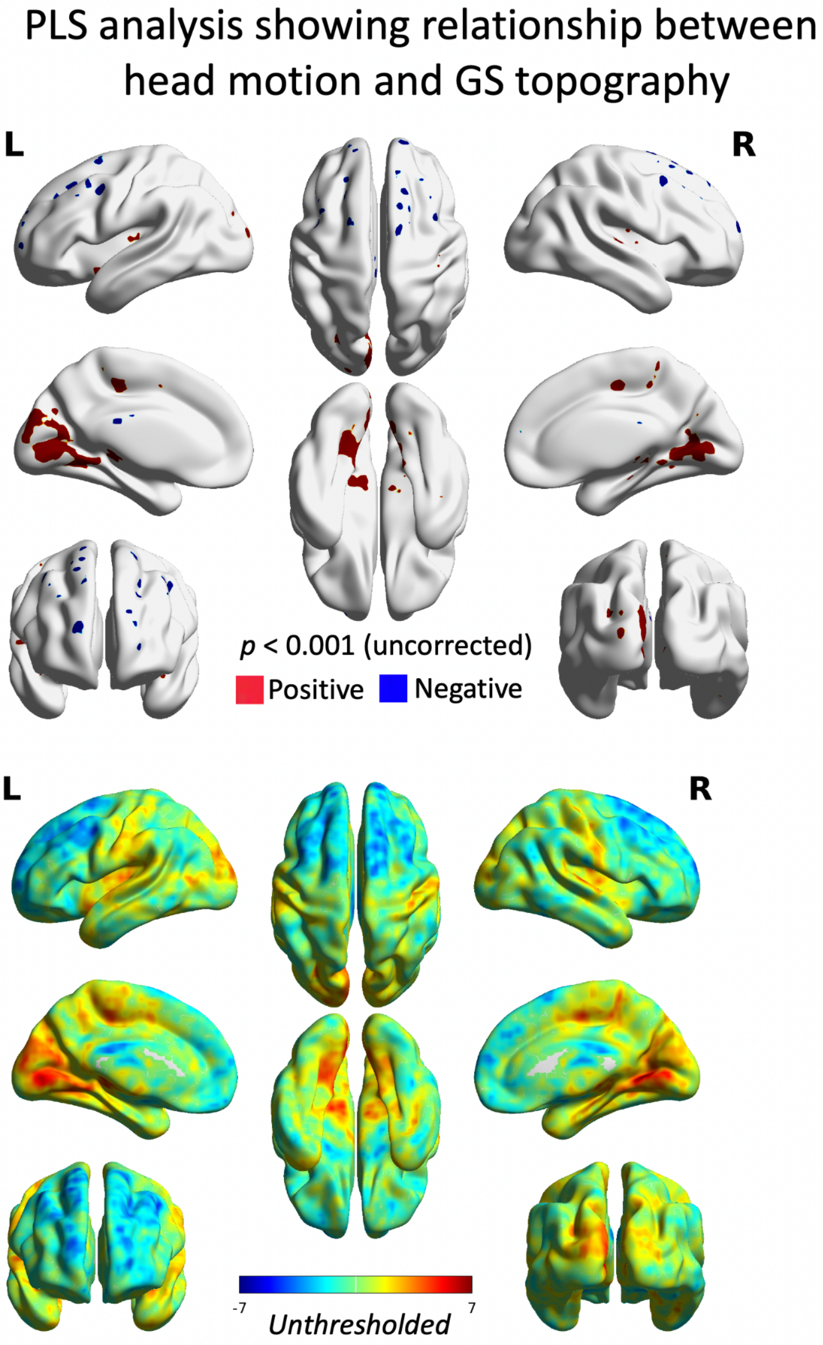

Supplemental Figure 15: Linear and quadratic GLM results for the relationship between GS topography and age when using an additional head motion covariate from a supplemental PLS analysis. The PLS head motion covariates account for the spatial representation of head motion within individual subject GS topography maps. These analyses show that the influence of head motion is represented in a spatially distinct manner in the GS topography maps when compared to the influence of age. The top panel are the same images from main Figure 3 and are presented here for comparison purposes.

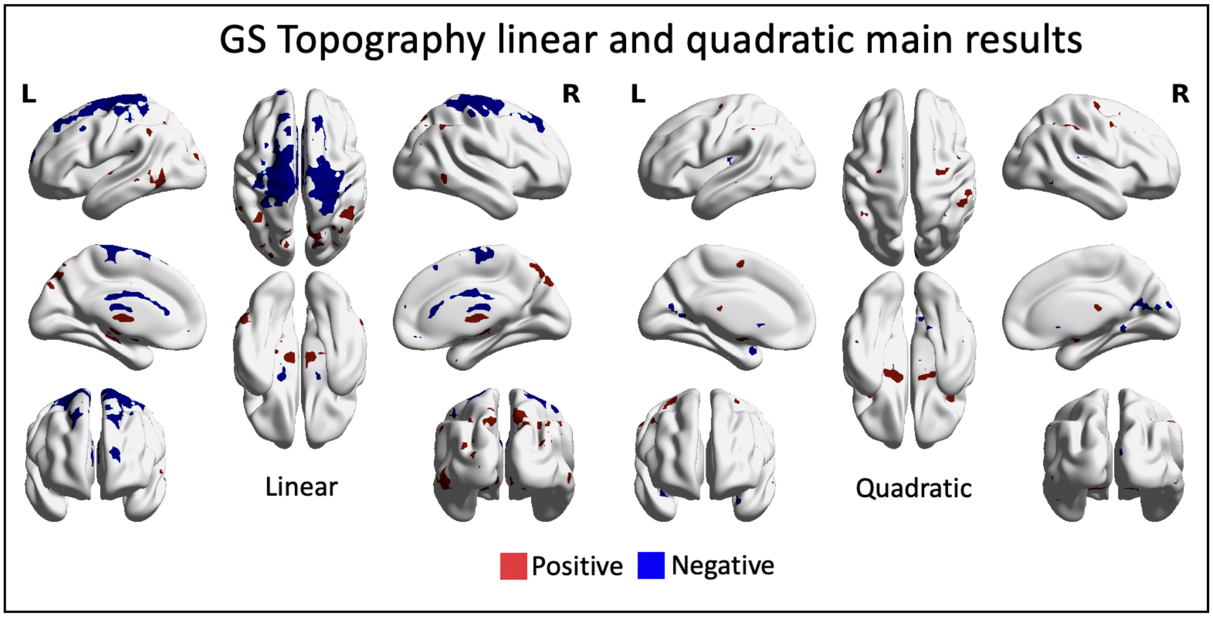

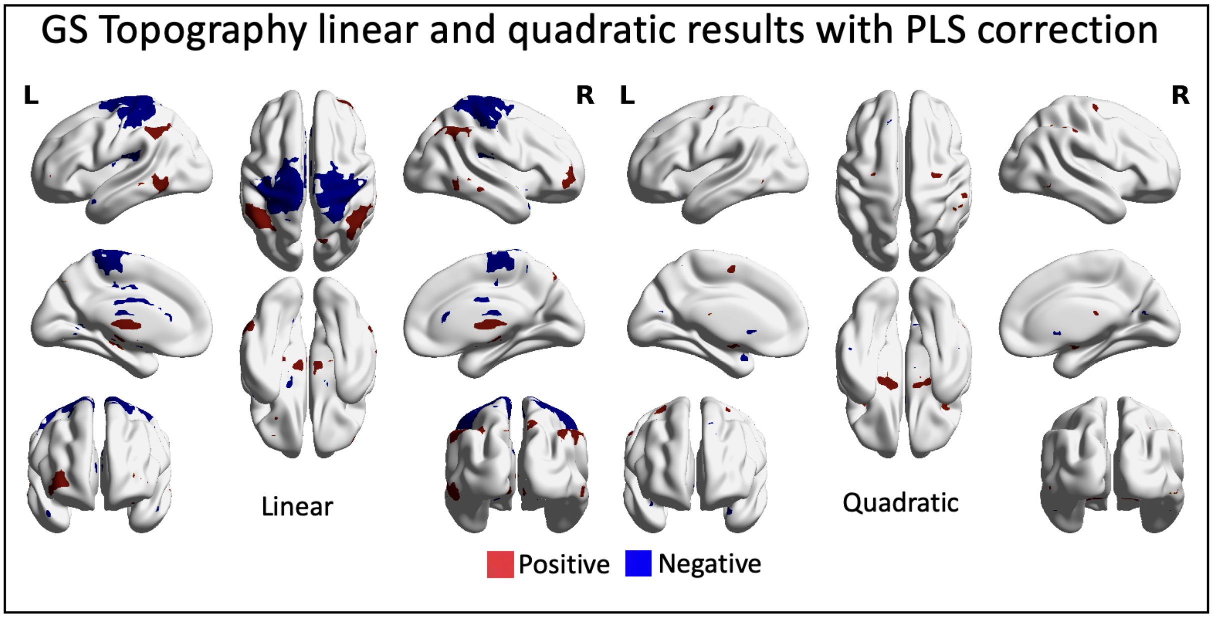

Supplement: Supplementary Material [file imag_a_00101-supp.pdf]
